# Personalized Computer Simulation of Diastolic Function in Heart Failure

**DOI:** 10.1016/j.gpb.2016.04.006

**Published:** 2016-07-29

**Authors:** Ali Amr, Elham Kayvanpour, Farbod Sedaghat-Hamedani, Tiziano Passerini, Viorel Mihalef, Alan Lai, Dominik Neumann, Bogdan Georgescu, Sebastian Buss, Derliz Mereles, Edgar Zitron, Andreas E. Posch, Maximilian Würstle, Tommaso Mansi, Hugo A. Katus, Benjamin Meder

**Affiliations:** 1 Institute for Cardiomyopathies, Department of Medicine III, University of Heidelberg, 69120 Heidelberg, Germany; 2 German Centre for Cardiovascular Research (DZHK), Heidelberg/Mannheim, Germany; 3 Siemens Healthcare, Medical Imaging Technologies, Princeton, NJ 08540, USA; 4 Siemens Healthcare, Strategy and Innovation, 91052 Erlangen, Germany

**Keywords:** Dilated cardiomyopathy, Tau, Myocardial stiffness, Computer-based 3D model, Personalized medicine, Diastolic function

## Abstract

The search for a parameter representing left ventricular relaxation from non-invasive and invasive diagnostic tools has been extensive, since heart failure (HF) with preserved ejection fraction (HF-pEF) is a global health problem. We explore here the feasibility using patient-specific cardiac computer modeling to capture diastolic parameters in patients suffering from different degrees of systolic HF. Fifty eight patients with idiopathic **dilated cardiomyopathy** have undergone thorough clinical evaluation, including cardiac magnetic resonance imaging (MRI), heart catheterization, echocardiography, and cardiac biomarker assessment. A previously-introduced framework for creating multi-scale patient-specific cardiac models has been applied on all these patients. Novel parameters, such as global stiffness factor and maximum left ventricular active stress, representing cardiac active and passive tissue properties have been computed for all patients. Invasive pressure measurements from heart catheterization were then used to evaluate ventricular relaxation using the time constant of isovolumic relaxation **Tau** (*τ*). Parameters from heart catheterization and the multi-scale model have been evaluated and compared to patient clinical presentation. The model parameter global stiffness factor, representing diastolic passive tissue properties, is correlated significantly across the patient population with *τ*. This study shows that multi-modal cardiac models can successfully capture **diastolic (dys) function**, a prerequisite for future clinical trials on HF-pEF.

## Introduction

The application of computational modeling to different organ systems has been gathering increasing interest from the research community. The possibility of performing *in silico* experiments on computer models that mimic patient’s organs has revved up the momentum of the evolution of virtual patient-specific models. The surge of interest has been driven by the prospect of being able to control all the variables to open up new possibilities toward better health care in a risk-free and ethically acceptable setting for the patient. The exponential growth of computational imaging capacities has also broadened the possibilities toward such models. From simplistic models based on geometric shapes as early as the 1960s to multi-scale multi-physics models, the transformation in this field has been tremendous [1], [2], [3], [4], [5], [6].

Heart failure (HF) remains the leading cause of death in developed countries [7], [8], [9]. The increasingly high incidence rates, hospitalization, and health expenditures compel a constant call for new strategies and progress in this field [10]. HF is a syndrome with diverse etiologies, characterized by the decline of cardiac systolic or diastolic function, resulting in insufficient blood supply to organs, organ dysfunction, and finally, failure [11], [12], [13].

A chronological retrospective analysis of HF therapy in patients with dilated cardiomyopathy (DCM) in the last century sheds light on difficulties in treating this disease. Expert guidelines currently outline HF therapy based on patients’ clinical presentation, cardiac systolic function, and specific biomarkers, but oversee, to some extent, the pathophysiology and etiology that lead to reduced cardiac function [13]. These rigid therapy regimes focus on relieving cardiac symptoms and tackle less the individual progression and the cause leading to this disease. Over the past three decades, drug therapy has undergone rapid progression in lowering the mortality and morbidity rates in HF patients [14]. The mortality rates of patients that present with progressed HF symptoms and receive optimal medical therapy remain high [14], [15]. Even the latest drug advancements present only a stepping stone toward the treatment of HF. The diversity of this disease, in its etiology and clinical presentation, suggests that the key to a better and cost-effective therapy is the individualized and personalized care. Personalized cardiac models have the potential in facilitating the achievement of this goal [16], [17].

The role of left ventricular (LV) systolic dysfunction has attracted broad attention from both clinical and experimental researchers [18], [19], [20], [21], [22], [23]. On the other hand, LV diastolic dysfunction has been relatively slow in gathering interest due to its complex role in the pathomechanism of HF [24], [25]. General consensus defines LV diastolic dysfunction as irregular cardiac functional relaxation, distensibility, and LV filling, which causes higher end diastolic left ventricular pressures [26]. To completely understand the pathogenesis of diastolic dysfunction, a broad appreciation of cardiac physiology in the diastole and its diverse compensation mechanisms is needed. Dyspnea, as a symptom of HF, is often attributed to diastolic dysfunction after exclusion of other probable causes [27], [28], [29], [30]. Its diagnosis remains a challenge in clinical settings because of the difficulties present in linearly quantifying the progression of this disease and assessing its significance to the patient [31]. The current non-invasive gold standard for the assessment of diastolic dysfunction remains the echocardiographic evaluation, especially Doppler measurements of transmitral flow and tissue Doppler imaging (TDI) [26].

The progress in the field of cardiac simulation has been on a rise in the last decade [32]. One of the first challenges in cardiac modeling is capturing the anatomical geometry of the heart. Simulating cardiac physical parameters relies heavily on ventricular geometry. Many of the early-proposed cardiac anatomical estimations were either based on geometrical models or post-mortem heart dissections. The first simplifications of the complex LV geometry have been based on spherical models [33]. Koushanpour and colleagues published one of the early simulations of LV dynamics based on spheroids in 1960s [34]. In this study, they compared the LV time course of tension using Laplace’s surface tension law in cats and turtles. Their findings highlighted the importance of cardiac size and shape in determining LV function. A gradual shift toward anatomical models, based on *ex vivo* human and animal hearts, could be observed, capturing a more accurate representation of cardiac anatomy [35], [36], [37].

Progress in other fields of science, especially in physics and mathematics, and advancements in computer technology opened up new possibilities toward improving existing computer simulations. The application of the finite element method in diverse sectors of engineering represented one of the major turning points in cardiac computational modeling and simulation. The conception and refinement of this method enabled the analysis of complex structural and mathematical problems [38], [39]. Janz et al. introduced one of the early cardiac mechanical models using the finite element method [40]. The cardiac model, in which the anatomical geometry is estimated from the hearts of Sprague–Dawley albino male rats, seemed to predict the gross free wall deformation with the assumption of an elastically linear and heterogeneous tissue [40]. Vinson et al. later described a human cardiac model using “36 brick type finite elements” representing the left ventricle [40]. As pointed out by the authors, one of the limiting factors at that time was “the capacity of the computer and computing time available” [41]. Today, current smart phones have more processing power than the computers used at that time.

The radical advances in cardiac imaging modalities and the implementation of non-invasive imaging sequences into the diagnostic algorithms marked the shift toward image-based models and allowed faster transition toward patient-specific cardiac models [42]. Most computational models to date selectively integrate elements (such as myocardial structure, structural pathologies, biomechanics, or electrophysiology) in various details and complexity, to suit the objective of the model [43].

We have proposed previously a patient-specific cardiac model that captures the biomechanical, hemodynamic, and electrophysiological cardiac functions in patients with DCM [2]. In this paper, we explore the feasibility of using such models to capture cardiac diastolic function in a similar patient population.

## Results and discussion

### Clinical characteristics of the patient population

**Figure 1:**
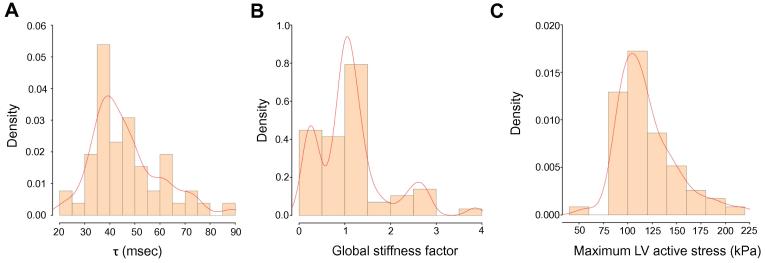
Distribution of the examined variables Distribution of the calculated time constant Tau (*τ*; **A**), global stiffness factor (**B**), and LV maximum active stress (**C**) across the study population is plotted. The brown bars represent the frequency density and the red lines represent the distribution curve overlay for each variable. LV, left ventricle.

A summary of the clinical parameters investigated in this study is presented in Table 1. The patients in our cohort are 54 years old on average. The majority of the recruited patients showed signs of HF with assessment of the New York Heart Association (NYHA) functional class II and III. The mean left ventricular ejection fraction (LV-EF) was 37%, with 5% of the recruited patients having an ejection fraction above 55%. HF drug therapy was initiated for all patients. The descriptive analysis of the invasive pressure measurements is presented in Table 2. As can be seen, the mean left ventricular end diastolic pressure (LV-EDP; mean 22 mmHg), the pulmonary capillary wedge pressure (PCWP; mean 20 mmHg), and the systolic pulmonary artery pressure (SAP; mean 40 mmHg) were all elevated as expected from the largely-symptomatic patient cohort. The calculated time constant Tau (*τ*) across the study population ranged 28–89 ms as shown in Figure 1**A**. Taking together the elevated pressure measured from the right circulation, approximately 40% of the patients proved to have a lengthened *τ* (duration >48 ms [44]), a sign of abnormal left ventricular relaxation.

### Simulation of cardiac parameters

**Figure 2:**
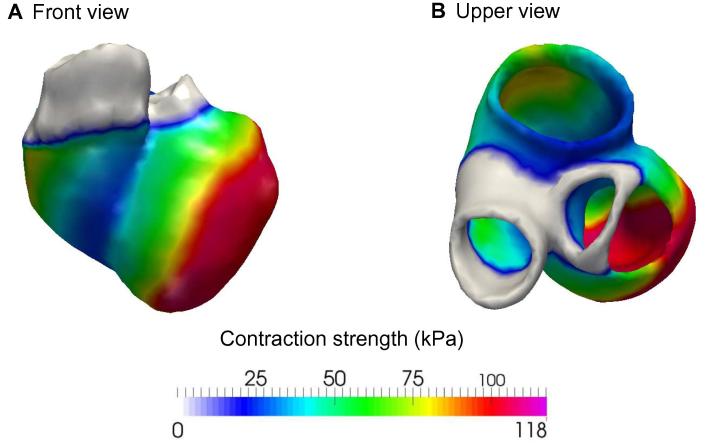
Map of the computed myocardium contraction strength in a patient-specific cardiac model The contraction strength is shown in the front view (**A**) and upper view (**B**) using color gradient with low intensity in blue and high intensity in red, non-contractile connective tissue is colored in gray.

The feasibility of using the presented cardiac model to capture cardiac systolic function in a clinical setting, in its strengths and limitations, has been previously reported [2]. In the present study, we aimed to examine how systolic and diastolic biomechanical parameters derived from the model, after completion of the fitting and personalization process, correspond to invasive and non-invasive clinical parameters of diastolic function. An example of a generated cardiac model of a patient in this study, after concluding the workflow algorithm, is shown in Figure 2. The systolic parameters, including computed LV-EF (cLV-EF; mean 35%), simulated stroke volume (sSV; mean 86 ml), maximum strength of active contraction (s0; mean 120 kPa), and global stiffness factor (HO factor; mean 1.1), are computed from the cardiac models for each patient as shown in Table 3. The distribution of global stiffness (HO factor) and LV maximum active stress (s0) across the study population is shown in Figure 1**B** and **C**, respectively.

### Assessment of the diastolic function

From early animal experiments investigating the maximal rate of pressure fall (max negative *dP*/*dt*) [45] to current echocardiographic TDI parameters in humans [46], the search for a parameter representing left ventricular relaxation from non-invasive and invasive diagnostic tools has been extensive [44]. The diastolic function of the heart is largely dependent on the passive myocardial properties, such as myocardial stiffness, which represents the effective elasticity of cardiac extra and intracellular composition. Preload, myocardial contractility, and regional dyssynchrony modulate myocardial relaxation [25]. The accurate characterization and assessment of diastolic dysfunction requires the simultaneous measurement of pressure and volume changes in the left ventricle during the diastole, which increases the complexity and difficulty of its precise clinical evaluation in living patients. Tau (*τ*), the time constant of isovolumic relaxation, is acknowledged as the time period needed for the ventricular pressure to fall to approximately 37% (or 1/e) of the pressure at the start of the isovolumic relaxation phase [47]. We used *τ* in this study, as a measure for the cardiac diastolic function, because *τ* remains a widely-accepted, less load-dependent surrogate for left ventricular relaxation and pressure decline [47], [48].

**Figure 3:**
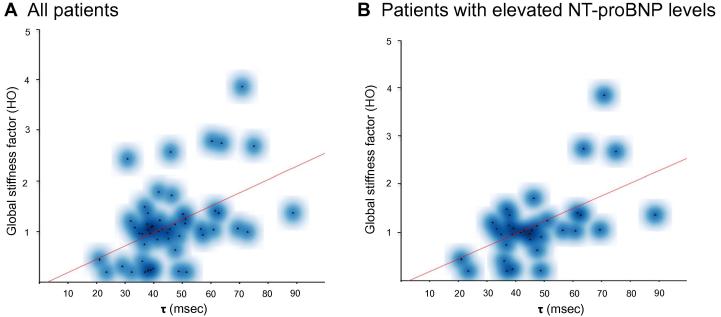
Correlation between the global stiffness factor and *τ* Scatter plots represent the correlation between global stiffness factor and *τ* across the study population for all patients (**A**) and in the subgroup for patients with elevated NT-proBNP levels (NT-proBNP levels >125 ng/l) (**B**). The blue clouds represent the frequency density. The red line represents the best-fit line for the correlation, which is generated using R. NT-proBNP, N-terminal pro-brain natriuretic peptide.

To assess the ability of the personalized cardiac model in capturing left ventricular relaxation, we correlated the model parameter of left ventricular global stiffness with *τ*. As presented in Table 4 and Figure 3**A**, there is a significant correlation (*P* = 4.1E−4) between the global stiffness factor and *τ*, whereas no significant correlation was found between left ventricular maximum active stress and *τ*. N-terminal pro-brain natriuretic peptide (NT-proBNP) is accepted as a prognostic biomarker in both systolic and diastolic HF [13], [49], [50]. We extended the analysis by subdividing the study population into patients with normal and elevated NT-proBNP plasma concentration (cut-off value of 125 ng/l). Interestingly, the correlation between global stiffness factor and *τ* was not only preserved but enhanced in the subpopulation with elevated NT-proBNP (125 ng/l) as shown in Table 4 and Figure 3**B**. The correlation between these two parameters was also preserved (*R* = 0.58, *P* < 0.05), with a higher cut-off level of 325 ng/l for NT-proBNP. At the same time, the correlation between LV maximum active stress, which represents the active and systolic component of myocardial contraction in the model, and *τ* remained non-significant. This observation underlines the potential benefit of combining molecular biomarkers with computational models.

Doppler echocardiography remains the current reference method for non-invasive assessment of diastolic LV function. Kasner et al. performed a clinical study evaluating the correlation between conventional or TDI echocardiographic diastolic indexes and pressure volume measurements from heart catheterization. E′ (early diastolic peak of the annular TDI measurements), E/E′ (ratio of transmitral flow and annular velocity), E′/A′ (ratio of early and late annular velocity) showed very modest correlations with *τ* of −0.33, 0.34, and −0.24, respectively [51]. Although the presented correlation between global stiffness factor and *τ* appears modest, it remains at least on the same level as those between *τ* and the echocardiographic parameters mentioned above.

## Conclusions

The clinical applicability of using *in silico* 3D computational cardiac models is promising, which strengthens the predilection toward its utilization in search of novel perspectives in risk stratification, therapy, and prognosis in other fields of cardiology [17]. The incentive toward the search for a better strategy to diagnose and evaluate diastolic dysfunction stems from the heterogeneity of results in clinical studies investigating HF with preserved EF (HF-PEF), with respect to mortality, quality of life, and cardiovascular risk [52]. The commonly-accepted consensus, which has prevailed over the years, remains that HF-PEF is associated with increased mortality and hospitalization [52], [53], [54]. As a diagnosis of exclusion for patients presenting with dyspnea and other HF symptoms, HF-PEF presents a challenge to physicians especially in an ambulatory setting. The differences in patient characteristics and demographics between patients with HF-PEF and those carrying HF with reduced EF (HF-REF) have raised further questions about the disease pathomechanism, severity, and clinical significance. In this study, we show that this personalized cardiac model can capture patient-specific diastolic parameters, which could hold the key toward solving difficult challenges in patients with HF-PEF.

More and more accurate and detailed models of cardiac function in both humans and animals have been abundantly reported, including biomechanical models that specifically investigate cardiac diastolic function [55], [56], [57], [58], [59]. However, few models integrate data from conventional standard clinical procedures to create a patient-specific electro-mechanical heart model. This study presents the feasibility of applying and integrating various experimentally-validated biophysical models to create a patient-specific multi-modal simulation of cardiac function in the diseased heart.

Our goal is the constant progression of the implementation of virtual cardiac models in a clinical setting to provide the patients with the optimal individualized medical care. Further advancement of computational modeling at different levels is anticipated in the near future. One of the first steps forward is validating the predictive prognostic power of such virtual models in a clinical setting. Secondly, capturing patient-specific cardiac fiber architecture remains one of the challenges and a limiting factor of advanced *in vivo* virtual models nowadays. The importance of fiber orientation in simulating cardiac electrophysiology and biomechanics has been abundantly described in previous studies [60] and diffusion tension MRI (DT-MRI) serves as a common approach to capture cardiac fiber orientation [61]. Due to technical difficulties present, like scan duration, myocardial respiratory displacement, and short transversal relaxation time, high resolution DT-MRI imaging was mainly utilized on explanted animal and human hearts. Algorithms for rule-based assignment of fiber orientation currently provide alternative to *in vivo* virtual models [62]. However, recent advances in cardiac DT-MRI render this approach feasible in the near future [63], opening up the possibility toward generating fully patient-specific myocardial fiber orientation and architecture. On another level, integrating not only parameters of cardiac electrophysiology but also histopathological myocardial structure and tissue specific passive physical parameters, like tensile strength, compaction and density of fibers, and fibrosis grade, from myocardial biopsies could be promising toward the complete *in silico* simulation of the individual heart.

## Materials and methods

### Patient population

Patients with HF symptoms were enrolled in this study after having given their written informed consent. Only patients receiving heart catheterization due to clinical necessity were included. To reflect broad representation of potential HF phenotypes, cases with slightly to severely reduced systolic function were included. Clinical evaluation, diagnostics, and follow-up were performed in adherence to hospital guidelines.

The enrolled patients underwent comprehensive clinical assessment constituting a detailed clinical history, physical examination, 12 lead electrocardiogram, echocardiography, 6 Minute Walk Test, spiroergometry, and comprehensive laboratory tests including NT-proBNP. For the clinical diagnostic process, patients underwent also procedures to ensure exclusion of secondary causes of DCM (left heart catheterization, cardiac MRI, extensive blood panel, and clinical history). Acute myocarditis, significant coronary artery disease (CAD), history of chemotherapy with cardio-toxic agents or chest radiation, valvular heart diseases, and probable secondary causes for DCM were exclusion criteria. A total number of *n* = 58 patients were investigated in this study.

### Hemodynamic data acquisition

Hemodynamic assessment was performed using left and right heart catheterization. All pressure curves were checked for calibration errors. The customary femoral access was used in all patients receiving simultaneous left and right circulation evaluation. Pressure measurements of the left ventricle and aorta were performed over repeated cardiac cycles prior to application of the contrast agent. Hemodynamic pressure analysis was performed using the computer-assisted software Metek (Roetgen, Germany). The intraventricular rate of change in pressure ((−) (+) *dP*/*dt*) was calculated during the procedure. Maximum values for (−) (+) *dP*/*dt* were identified and output for each cardiac cycle. The calculation of *τ* (time constant of isovolumic relaxation) was based on the approach described by Weiss and colleagues [64], which assumes an exponential decline in left ventricular pressure during the isovolumic time period. *P*(*t*) = *P*(*t* = 0) x e−*t*/*τ* and *τ* = −*P*/(*dP*/*dt*).

### MR data acquisition

To further evaluate the clinical phenotype, all patients underwent cardiac MRI analyses (1.5T cMRI, 32Ch RF platform, Philips Achieva). Standard multi-slice 2D steady-state free precession sequences (SSFP), late gadolinium enhancement (LGE) multi-slice inversion recovery sequence, and feature tracking imaging were included in the procedure protocols. Comprehensive 2D echocardiographic assessment of systolic and diastolic function according to current guidelines and hospital standards was also performed in all patients.

### Personalized cardiac model in patients with dilated cardiomyopathy

**Figure 4:**
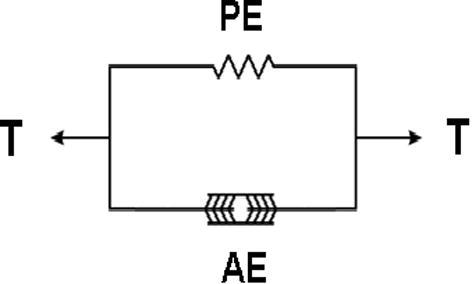
Schematic representation of the classical Hill’s muscle model The model has two parallel components: an active (AE) and a passive (PE) component. The total stress produced by the tissue is indicated by T.

The computational workflow and process of simulating the personalized multi-scale multi-physics model based on the acquired clinical data has been thoroughly described previously [2]. We briefly recall here the model assumptions related more specifically to the description of cardiac biomechanics. We adopt the Hill–Maxwell framework to represent the interplay between active contraction and passive response of the myocardium [65] (Figure 4).

The myocyte contraction is modeled following the approach presented by Sermesant and colleagues, for which the contraction is related to the action potential through a bi-exponential law [66]. We parameterize this law by the maximum strength of active contraction (s0), the rate of contraction (the speed at which the tissue contracts during depolarization), and the rate of relaxation (the speed at which the tissue relaxes during repolarization). The passive response of the myocardium to mechanical stress is described by the non-linear, hyper-elastic and orthotropic tissue model proposed by Holzapfel and colleagues [67]. We consider a global scaling factor (HO factor) for the reference model parameters provided by Holzapfel and colleagues, offering a lumped representation of the tissue stiffness [67]. The electromechanical model provides computed cardiac dynamics, from which we extract simulated ejection fraction as the clinical parameter of interest. More details on the personalized cardiac model can be found in the references cited in this section.

### Statistical analysis

The statistical analysis was performed using the conventional “R” software (Version 3.2.2). The parameters *τ*, global stiffness factor, and LV active force are continuous and show an approximate normal distribution. Therefore, a linear correlation analysis using Pearson’s correlation coefficient through the “cor” and the “cor.test” function was applied. The parametric *P* value, with a significance level of 0.05, was computed for all performed correlations. To account for a possible non-linear relationship between *τ* and global stiffness factor, a logarithmic analysis of both parameters is also presented (Table S1). A possible monotonic correlation was analyzed using the Spearman rank correlation method. The results obtained were similar but non-superior to those based on the linear correlation analysis and were not presented in the current study to avoid repetition. Histograms were calculated using the “hist” function with standard parameters. In order to visualize the output, scatter plots were generated for the significant correlations. Smoothing of scatter plots was carried out by the “smoothScatter” function.

## Authors’ contributions

BM, AA, EK, FS, and TM designed the study; AA, FS, HK, EK, BM, DM, EZ, and SB carried out patient data acquisition; TM, TP, VM, DN, BG, AEP and MW performed the computational analysis, and AL, AA, BM, FS, and EK carried out statistical analysis. AA, EK, BM, TM, TP, DM, and HK were involved in manuscript drafting and revision. All authors read and approved the final manuscript.

## Competing interests

This work was in part conducted within an industry supported project (Siemens Healthcare, Siemens Research Project). TP, VM, DN, BG, AEP, MW, and TM are employees of Siemens Healthcare. There are no further conflicts of interest. The features mentioned herein are based on research, and are not commercially available. Its future availability cannot be guaranteed due to regulatory reasons.

## Figures and Tables

**Table 1:** Clinical characteristics of the recruited patients

**Patient characteristics**	**Value**
Age, mean ± SD, year	53.7 ± 12.6
Age at onset ± SD, year	52.8 ± 12.8
BMI, mean ± SD, kg/m^2^	27 ± 5.6
Heart rate, mean ± SD, beats/min	78 ± 20
Blood pressure, mean ± SD, mmHg	
Systolic, mmHg	122 ± 17
Diastolic, mmHg	77 ± 11
Diabetes, number (%)	9 (19%)
Left bundle-branch block, number (%)	13 (22%)
Atrial fibrillation, number (%)	9 (16%)
6MWT, mean ± SD, m	511 ± 120
Dyspnoea, number (%)	
NYHA I	11 (19%)
NYHA II	28 (48%)
NYHA III	17 (30%)
NYHA IV	2 (3%)
Family history of SCD or DCM, number (%)	11 (19%)

*Laboratory tests*	
White blood cell count, mean ± SD, /nl	7.8 ± 2.4
Hemoglobin, mean ± SD, g/dl	14.4 ± 1.5
eGFR, mean ± SD, ml/min/1.73 m^2^	88.6 ± 16.3
Creatinine, mean ± SD, mg/dl	0.9 ± 0.2
NT-proBNP, median (1Q;3Q), ng/l	767 (104;2385)
hs-TNT, median (1Q;3Q), pg/ml	16(8;38)
Medications, number (%)	
Aspirin	20 (36%)
ß-blocker	54 (93%)
ACE inhibitor or ARB	58 (100%)
Loop diuretic	30 (54%)
Aldosterone antagonist	35 (60%)
Statin	24 (44%)
Digoxin	7 (12%)

*Echocardiography*	
LV ejection fraction, mean ± SD, %	32 ± 15
LV-EDD, mean ± SD, mm/m^2^	57 ± 9
LV-ESD, mean ± SD, mm/m^2^	43 ± 13

*MRI*	
LV ejection fraction, mean ± SD,%	37 ± 15
LV stroke volume, mean ± SD, ml	84 ± 28
LV-ESV index, mean ± SD, ml/m^2^	85 ± 57
LV-EDV index, mean ± SD, ml/m^2^	130 ± 54
LV-ESD index, mean ± SD, mm/m^2^	26 ± 7
LV-EDD index, mean ± SD, mm/m^2^	31 ± 5
LV mass index, mean ± SD, g/m^2^	59 ± 21

*Note:* 6MWT, 6 Minute Walk Test; NYHA, New York Heart Association functional classification; SCD, sudden cardiac death; DCM, dilated cardiomyopathy; eGFR, estimated glomerular filtration rate; NT-proBNP, N-terminal prohormone of brain natriuretic peptide; hs-TNT, high sensitive troponin T; ACE, angiotensin-converting-enzyme; ARB, angiotensin II receptor blocker; LV, left ventricular; EDD, end diastolic diameter; ESD, end systolic diameter; MRI, magnetic resonance imaging; ESV, end systolic volume; EDV, end diastolic volume.

**Table 2:** Summary of invasive pressure measurements and calculations

**Parameter**	**Value**
Left ventricular end-diastolic pressure, mean ± SD, mmHg	22 ± 8.8
Pulmonary capillary wedge pressure, mean ± SD, mmHg	20 ± 9.1
Mean pulmonary artery pressure, mean ± SD, mmHg	28 ± 11.2
Systolic pulmonary artery pressure, mean ± SD, mmHg	40 ± 13.4
(−)*dP*/*dt*(max), mean ± SD, mmHg/s	1381 ± 404
(+)*dP*/*dt*(max), mean ± SD, mmHg/s	1306 ± 488
Tau (*τ*), mean ± SD, ms	49 ± 13.3

**Table 3:** Summary of the simulated parameters from the personalized model

**Parameter**	**Value**
Global stiffness factor, mean ± SD, no unit	1.1 ± 0.73
Left ventricular maximum active stress, mean ± SD, kPa	120 ± 30.3
Simulated stroke volume, mean ± SD, ml	86 ± 27.2
Computed left ventricular ejection fraction, mean ± SD,%	35 ± 13.6
<30, number (%)	23 (39.6)
30–44, number (%)	19 (32.7)
45–54, number (%)	13 (22.4)
⩾55, number (%)	3 (5.2)

**Table 4:** Statistical analysis of the correlations between the simulated systolic and diastolic parameters with Tau in patients

**Patient group**	**Correlation of Tau (*τ*) with global stiffness factor**		**Correlation of Tau (*τ*) with LV maximum active stress**	
***R* value**	***P* value**	***R* value**	***P* value**
All patients	0.47	4.1e−4	−0.23	9.8e−2
Patients with elevated NT-proBNP (>125 ng/l)	0.59	2.4e−4	−0.17	3.4e−1

*Note:* NT-proBNP, N-terminal pro-brain natriuretic peptide.
