# Supplementary material for: Personalized Computer Simulation of Diastolic Function in Heart Failure
Source: Genomics Proteomics Bioinformatics. 2016 Jul 29;14(4):244–52. doi: 10.1016/j.gpb.2016.04.006 (PMC4996856; doi:10.1016/j.gpb.2016.04.006)
Supplement: Supplementary Table S1 [file mmc1.docx]

**Table S1 Summary of the linear and logarithmic statistical analysis of the correlations between τ and the simulated biomechanical model parameters**

| **Linear model** | **R value for correlation with τ** | ***P* value** | **Logarithmic model** | **R value for correlation with log (τ)** | ***P* value** |
| --- | --- | --- | --- | --- | --- |
| **Global stiffness factor** | 0.47 | 4.1e−4 | **Log (global stiffness factor)** | 0.46 | 5.2e−4 |
| **LV maximum active stress** | −0.23 | 9.8e−2 | **Log (LV maximum active stress)** | −0.22 | 1.2e−1 |
| **Simulated SV** | −0.13 | 3.5e−1 | **Simulated SV** | −0.14 | 3.4e−1 |
| **Computed LV-EF** | −0.31 | 5.1e−4 | **Computed LV-EF** | −0.32 | 3.3e−4 |

*Note:* LV, left ventricle; LV-EF, left ventricular ejection fraction; SV, stroke volume.
